# Implementing FCTC Article 17 Through Participatory Research With Bidi Workers in Tamil Nadu, India

**DOI:** 10.1093/ntr/ntac075

**Published:** 2022-04-28

**Authors:** Andrew Russell, P Chandra, Mary Robson, Pradeep Narayanan, Stanley Joseph, Pavan Mukherjee, Mira Aghi, Marty Otañez, Madhumita Dutta, Upendra Bhojani, Prishanti Pathak, Sushil John

**Affiliations:** Department of Anthropology, Durham University, Durham, UK; D Arul Selvi Rehabilitation Trust, Tirupattur, India; Institute for Medical Humanities, Durham University, Durham, UK; Praxis Institute for Participatory Practices, New Delhi, India; Praxis Institute for Participatory Practices, New Delhi, India; Low Cost Effective Care Unit, CMC Vellore, Vellore, India; Healis Sekhsaria Institute for Public Health, Mumbai, India; Anthropology Department, University of Colorado, Denver, CO, USA; Department of Geography, Ohio State University, Columbus, OH, USA; Institute of Public Health, Bengalaru, India; Department of English Studies, Durham University, Durham, UK; Low Cost Effective Care Unit, CMC Vellore, Vellore, India

## Abstract

**Introduction:**

The exploitation, poor conditions, and precarity in the bidi (hand-rolled leaf cigarette) industry in India make it ripe for the application of the FCTC’s Article 17, “Provision of support for economically viable alternative activities”. “Bottom-up”, participatory approaches give scope to explore bidi rollers’ own circumstances, experiences, and aspirations.

**Methods:**

A team of six community health volunteers using a participatory research orientation developed a questionnaire-based semi-structured interview tool. Forty-six bidi rolling women were interviewed by pairs of volunteers in two northern Tamil Nadu cities. Two follow-up focus groups were also held. A panel of 11 bidi rollers attended a workshop at which the findings from the interviews and focus groups were presented, further significant points were made and possible alternatives to bidi rolling were discussed.

**Results:**

Bidi workers are aware of the adverse impact of their occupation on them and their families, as well as the major risks posed by the product itself for the health of consumers. However, they need alternative livelihoods that offer equivalent remuneration, convenience, and (in some cases) dignity. Alternative livelihoods, and campaigns for better rights for bidi workers while they remain in the industry, serve to undercut industry arguments against tobacco control. Responses need to be diverse and specific to local situations, i.e. “bottom-up” as much as “top-down”, which can make the issue of scaling up problematic.

**Conclusion:**

Participatory approaches involving bidi workers themselves in discussions about their circumstances and aspirations have opened up new possibilities for alternative livelihoods to tobacco.

**Implications:**

Progress with the FCTC’s Article 17 has generally been slow and has focussed on tobacco cultivation rather than later stages in the production process. The bidi industry in India is ripe for the application of an alternative livelihoods approach. This study is one of the first to use participatory methods to investigate the circumstances, experiences, and aspirations of bidi workers themselves.

## Introduction

This article reports a new direction in alternative livelihood work with bidi (leaf cigarette) workers in two cities in Tamil Nadu, Southern India. It first addresses the issue of why the FCTC’s Article 17 “Provision of support for economically viable alternative activities” has been insufficiently implemented to date, challenging its predominant focus on tobacco cultivation. There is a need to address manufacturing further up the supply chain and to attend to forms of tobacco production other than those of the transnational tobacco corporations (TTCs), such as the bidi industry in Southern India.

Few of the 181 Parties to the FCTC seem to take their obligations on Article 17 seriously. Where efforts have been made to discourage the manufacturing of tobacco products, these have generally been unsuccessful. Alternative livelihoods are inherently complex and there is a “scarcity of information on specific programs and relevant research”.^1^ Article 17 can be criticized for taking what is essentially a “top-down” approach to alternative livelihoods. This means the circumstances, experiences, and aspirations of people in tobacco-dependent livelihoods are often insufficiently considered. There are structural problems with FCTC processes and powerful TTCs can derail alternative livelihood discussions or co-opt them in the interests of spurious corporate social responsibility. For all these reasons, Article 17 has been relatively poorly implemented worldwide.

Bidi industry production methods in India are different to those of the cigarettes produced by the TTCs.^2^ Bidi (or “beedi” or “biri”), “a leaf-rolled cigarette made of coarse, uncured tobacco, tied with a string at one end”,^3^ is smoked by 7.7% (71.8 m) of the Indian adult population compared to the 4% (37.0m) who use cigarettes.^4^ Ninety-five percent (68.2 m) of bidi-smokers are men. Bidi manufacturing is predominantly a “cottage industry”, with its manufacturing base in people’s homes. The Beedi and Cigar (Conditions of Employment) Act 1966 led to outsourcing so that bidi company owners could avoid the legal requirements regarding working hours, paid holiday, annual and maternity leave it introduced.^5^ An estimated 90% of bidi production is by women.^6^ Children as young as five or six are also frequently involved.^7,8^ The bidi industry was previously largely confined to registered companies in the organized sector but over the past 50 years has shifted to unregistered companies in the informal sector, largely to avoid labor regulations and taxation.^9^ Bidi rollers in the unregistered sector (around 90% of the total, an estimated 2.9 m in 2010–2011)^10^ are indirectly employed through a system of contractors and sub-contractors in order to keep unit sizes and hence the tax burden for owners (the “bidi barons”) low. Profits in the bidi industry have risen substantially in recent decades, while the wages of bidi rollers in the unregistered sector have decreased.^9^

Bidi rolling is an exploitative source of income^11,12^ across South Asia^13^ and is ripe for the application of an alternative livelihoods approach.^14^ Apart from the great long-term harm to human health from the commodity produced,^15^ bidi rolling itself is an unhealthy and discriminatory occupation.^16,17,18^ It negatively impacts women’s education, while dust inhalation and a hunched work posture are detrimental to their health.^6,19^ However the health problems of bidi rolling are hard to disaggregate from the diseases of poverty more generally. Piece rate wages for bidi rolling vary across India. In Tamil Nadu (TN), where the study to be reported was undertaken, they are generally about US$2 for 1000 bidis compared to an average daily wage rate for unskilled labor in the state of nearly US$6. According to Rout et al “workers continue in this informal industry despite poor wages, exploitation by middlemen, absence of social security and adverse impact on health simply because they have no alternative”.^20^ With some notable exceptions,^21,22,23^ the voices of bidi industry workers concerning their current situation, the occupational and public health problems they face and the potential for alternative livelihoods, are rarely heard.

For the past fifteen years staff in the Low Cost Effective Care Unit (LCECU) at Christian Medical College (CMC), Vellore, have undertaken outreach work with disenfranchised communities in and around the city, many of whom are bidi rollers. Vellore is a major center for bidi production in TN, and TN is the second largest state in India with regards to numbers of bidi workers, outputs, and sales volumes.^21^ The LCECU was focused on clinical medicine administered to individual patients and their families, and time and resources made it difficult to address the wider determinants of health.^24^ With an inclusive and holistic approach to alternative livelihoods for bidi rollers, this project explored the potential for participatory methods to better align policy initiatives such as the FCTC’s Article 17 with local people’s circumstances, concerns, and aspirations.

## Methods

Our methodological orientation was participatory action research (PAR), which challenges and disrupts the power relationships of more traditional research methods by blurring the boundaries between researchers and researched.^25,26^ Arnstein’s “ladder of participation”^27^ represents participation on a scale from “consultation” to “devolved power”. Staff at Praxis, the Institute for Participatory Practices, New Delhi, who led the PAR approach of this project, work at the upper end of Arnstein’s ladder. Efforts to devolve power, in this case, are reflected in (1) the involvement of two former bidi workers in critical discussion of how the research tools were designed and the outputs analyzed, (2) the presentation of findings to a panel of workers who provided their feedback and reflections; and (3) using the findings to facilitate a local intervention on livelihoods. The D Arul Selvi Rehabilitation Trust, an NGO in Tirupattur, northern TN, offered the support of six of its women community health volunteers (CHVs) in conducting this study. Some, but not all, of these women came from bidi rolling families.

Praxis staff worked with these women on a “five-tool system” to find out more about the lives and aspirations of bidi rollers in Tirupattur and Vellore. The five sections were (1) “Who am I”, (2) “About bidi work”, (3) “Life history tool”, (4) “Reason tool—why I stay in bidi work”, and (5) “If I have tried to quit bidi work—what happened?” A lot of this was done diagrammatically using sticky labels and a lifeline to chart the ups and downs of people’s lives.

The volunteers, working in pairs, used this system to develop a questionnaire-based semi-structured interview tool. After pilot testing, they started data collection. Selection of participants took place by visiting hamlets and informal settlements where bidi rolling was known to be common. Purposive sampling of bidi workers willing to share their viewpoints in these settlements led to 24 interviews in Tirupattur and 22 in Vellore, in June and July 2019. Interviews took place in the bidi workers’ homes and typically took between 20 and 40 minutes. Invitations into homes was a gesture of trust in the project. Sometimes the interviews took place in private although more often one or family members and/or neighbors were present, which may have influenced the comments participants felt able to make. One of the volunteers took notes during the interview which they cross-checked with their fellow interviewer afterwards. Notes were translated from Tamil into English and written up as individual case histories in Microsoft Word. These case histories were independently coded (by AR and PC) and subject to content analysis. The themes identified followed the orientation of the five-tool system, viz. life circumstances, experiences of bidi work (both negative and positive), experiences of seeking alternatives, and aspirations for the future.

Women who participated were invited to attend a focus group (FG) with their peers. Five refused to participate for fear of losing their jobs or being subject to harassment, although some also cited daily household and work schedules as a reason for not participating. The FGs took place in September 2019 following volunteer training from Praxis. One of the CHVs acted as moderator and two as recorders. The FG held at CMC Shell Hospital, Vellore, involved six bidi rollers. The other, held at the D Arul Selvi Trust founder’s residence in Tirupattur, was attended by 14 bidi rollers. The FGs each took roughly one hour and were recorded using audio recorders. These recordings were translated into English and transcribed in Microsoft Word. Findings from the FGs have been incorporated into the results which follow, as are some basic descriptive statistical analyses of the participants.

The results were shared at a three-day workshop involving research partners, collaborators, 11 bidi rollers, and members of the wider tobacco control community held at CMC Vellore in December 2019. Interactions were supported by a creative facilitator (MR) from the Institute for Medical Humanities at Durham University. She led participants in a group work exercise that generated a set of values and intentions in response to the question “how will we best work together?” Answers included the need for inclusivity, active listening, and overcoming language barriers. Written down in English on a flip chart and orally translated into Tamil, these agreed ways of working were recited as a collective litany each morning.

Ethical approval for the study was received from Durham University’s Research Ethics Committee (UK) and the Institutional Review Board at CMC Vellore (India).

## Results

### Circumstances

The average age of participants in the study was 41 (median 40), although participants ranged in age from 16 to 65. In Indian government classificatory terms, 20% were from scheduled castes and 75% were from other backward classes, i.e. all communities that have been historically marginalized in India and face continued oppression and social, economic, and educational isolation. Two-thirds were Hindu and one-third Muslim, reflecting the higher proportion of Muslim women working in the bidi industry compared to the rest of the population.

Forty percent of the women had not attended school; only 10% had reached 10^th^ standard (i.e. 10 years of education). More than half the participants were second-generation bidi rollers—25% were first generation. The majority had been rolling bidis for 15–20 years.

### Negative Aspects of Bidi Work

Working hours were an issue for 83% of the women. Statements like “I work for 12 hours and earn Rs 170 [c. US$ 2.25] per day”, and “I am forced to labor into the night to complete the work”, were typical. The long hours and poor remuneration were frequently mentioned, as was the need to do domestic chores, care for children and prepare meals in a manner akin to the “second shift”.^28^ “We have to roll large numbers of bidis—if the number is reduced, the pay is reduced. There is no provision for leave, continuous daily work is expected”, said one woman. Yet because the bidi industry was declining, some found getting full-time work within it increasingly difficult.

Eighty percent of participants had health problems. One described “dizziness and pain in my hands, legs, and hips. Diabetes and BP [blood pressure] detected”. Sometimes health problems were compounded by the lack of protective equipment. “Sharp bits of tobacco prick our fingers and cause injury. My mother lost a finger”, one explained.

Entrapment was a common motif. “I am trapped in this work as I do not know anything else. I am really helpless”, said one. Another explained, “I cannot leave this work because I am disabled and have some health problems. My coworkers advise me against leaving this job”. Lack of education was another common refrain in the entrapment narrative. One woman felt “trapped in this job because I started at a very young age and did not study. I do not know of any other job”. However reaching 10^th^ standard was no guarantee of better work. One participant recounted: “I discontinued education in my 10^th^ class [and] I learnt and started working as a bidi worker. Had I continued my studies, I could have gone for better work”. Children engaged in bidi work also experienced negative educational consequences. “My children’s education is affected because they have to help us in tasks such as leaf preparation, tying threads etc.”, one mother explained.

Family circumstances also played a significant role in starting bidi work and then feeling trapped by it. One woman explained that her father’s health was bad and he was addicted to alcohol. “I learnt this profession very early in life and have always regretted it”, she said. Alcohol appeared a key player in other narratives too. According to one participant, “my girls discontinued their studies as it is difficult for me doing bidi work. I do the work and my husband grabs the pay and buys alcohol”. Kinship was invoked in other ways too. “I was forced to learn the job as my mother-in-law scolded me”, said one woman, while another, a widow, explained how she learned bidi work from her parents. “The contractor is also a relative”, she added. Peer influence was also strong. “All my neighbors are doing this work, so I started doing it”, said one woman.

### Positive Aspects of Bidi Work

Positive viewpoints generally concerned the convenience of home-based work or the financial benefits of bidi rolling (notwithstanding the low wages). “I am able to do household work and look after children while rolling bidis at home. It is a protective environment and I feel safe while at work”, said one woman. Other comments included “we do bidi work as and when we want”; “there is no need of travel—time is saved”; “we need not go out in the rain or sun seeking work”; and “in our community women are not supposed go out, hence doing the work at home is good”.

As for the financial benefits, one woman claimed bidi rolling was “the only job that provides a regular/steady income”. For another, “in the case of other types of work, some days we might be jobless. But bidi rolling allows us to work and earn daily”. One woman appreciated how “when the work is done we get immediate payment … other jobs delay payment”.

Women appreciated having their own money and, in some cases, autonomy over how it was spent. Further, for one woman, with “economic independence [comes] … self-respect. The community respects me”. Another claimed she “was able to look after my children, educate them, get the girls married and manage the medical expenses for my husband’s illness. I have also bought jewels”. Girls born into bidi-rolling households may have better marriage prospects because a bidi-rolling family looks for a daughter-in-law who is ready to roll bidis as soon as she gets married.^29^ Further financial benefits came through access to loans. “It is easy to get loans and pay them back because of the steady income”, said one woman. For some, bidi work gave the chance for educational advancement too. “I am doing part-time bidi work in the evening to meet my educational expenses. No other agency other than the bidi company is offering part-time work like this. It also offers protection from sexual abuse and physical harassment”.

### Staying Put

Phrases about bidi rolling like “it has helped my family so far”, and “this is the only job that gives me an income currently” were common. The nature of the work (compared to other options) was sometimes significant. One woman explained, “I cannot do heavy work; other work is heavy with fixed timings”. For another, “because I am able to manage my household and look after the children with this job, there is no need to look out for another job”.

Yet, despite the homeworking and financial benefits expressed by some, 85% of participants wanted to leave bidi work and many would have been happy not to have started. “I do not have any other skill due to my lack of education. If I had studied, I would have got a better job”, said one woman. Some had attempted to leave, but circumstances prevented this being a sustainable move. “I tried to get alternative work but I continue [bidi rolling] because I was unsuccessful”, said one woman. Others put financial or temporal restrictions on the alternative employment they would be willing to take, such as “if there was any other job which gave me a steady income of Rs. 200–300 per day, I would willingly leave bidi rolling”, and “if a job with the same income was available between 8 am and 5 pm, I would take it up”.

### Seeking Alternatives

Where women had moved into alternative livelihoods, tailoring, flower selling, small shop work, and shoe factories were the four main options. A former bidi roller who had become a flower vendor considered it “a better job”. Another had given up bidi work to become a tailor, and found she made more money at it. One had “tried to work in a fancy store [but] as I could not take care of my children, I gave it up and came back to the bidi industry”. Starting a new business was challenging. “I am interested … but there are no adequate funds available”, said one woman. Sometimes women combined different jobs. One participant explained “I rolled bidis for 10 years. I now work in a shoe company and as a part-time tailor. I earn Rs 4160 [US$ 56] per month. I have got a better job with better income [and] I am aware of developments and events outside my house”. Bidi rolling might remain part of the combination. “I am trying but cannot leave it entirely”, said one woman; “I have taken up house-maid work in an attempt to escape it totally. However, I need the wages from bidi work for daily expenditure”.

### “It is our Fate”

For one participant “bidi work appears to be a good work but it is harmful. We are unable to complain, it is our fate”. Despite this, over 80% were adamant that the job should not continue with the next generation, despite the pressures to involve their children in the business. Statements like “I won’t involve my children in this work”, and “I want the work to end with my generation in the family”, were common.

## Discussion

The workers’ panel at the December 2019 final workshop in Vellore weighed up the positives and negatives of bidi work and made five significant points: (1) workers know the adverse health impact of bidi rolling on them and their families, especially children; (2) workers still make this choice because some kind of livelihood is of primary importance to them in order to fulfill all other needs; (3) such work also brings dignity to women in otherwise unbalanced household power relationships; (4) workers have tried alternative options and were either forced back into bidi work due to contractors’ deceit or returned voluntarily because other options were less convenient, unreliable, or more poorly remunerated; (5) that while they agree about the need for tobacco control and acknowledge bidi work promotes tobacco, their livelihood needs outweigh any moral or public health imperative to stop.

The intersections of these three domains—health, livelihood, and dignity—means that there is no single “bidi workers’ voice”. Rather, bidi work is situated on a spectrum. At one extreme is the view that bidi work provides a regular livelihood, the opportunity to meet other needs, and the greater chance of intra-household respect. At the other, bidi work is intrinsically detrimental to health, an exploitative livelihood that denies women a decent wage while reinforcing patriarchy and the domestic ghettoization of marginalized women. Policymakers need to decide which of these extremes should inform policy interventions and the formulation of schemes and programs. While bidi work is a form of tobacco capitalism,^30^ it is structured and organized very differently to the TTCs most global tobacco control efforts. Health and livelihood issues in bidi work lie in the realm of solutions, but the issue of dignity remains in the realm of problems since there are rarely effective efforts to engage with it. More generally, global tobacco control needs to engage better with postcultivation stages of production, taking issues like dignity and fairness seriously. Only through these means will alternative livelihood interventions be formed that have the chance of scaling up.

Limitations to our study include the potential that the women involved said what they thought researchers wanted to hear rather than what they truly believe. However, having Tamil-speaking female health volunteers from the same communities as researchers helped to mitigate against this possibility. Results are specific to the two research sites and are not necessarily generalizable to the rest of Tamil Nadu or India, although we would argue that knowledge and interventions should be diverse and specific to local situations, i.e. bottom-up as much as top-down. While the bidi industry is not presently obstructing alternative livelihoods to the extent that corporate tobacco does, bidi workers should consider ways to self-organize and counter any resistance from bidi companies. Alternative livelihoods and campaigns for better pay, conditions, and equipment for bidi rollers, while they remain in the bidi industry, serve to undercut industry claims that bidi manufacturing makes a significant contribution to the national economy,^31^ claims used to forestall effective tobacco control efforts.^9^ Participatory approaches, connecting local level experiences of labor exploitation with state, national and global level perspectives, give bidi rollers themselves a stake in the policy decisions that affect their lives and futures.

## Conclusion

Implementation of the FCTC’s Article 17 has generally been slow and has focussed on tobacco cultivation rather than later stages of the production process. Through participatory research and action with bidi workers in two northern Tamil Nadu cities, this article has highlighted both negative and positive perspectives on bidi rolling. We have seen how there is not one “bidi workers’ voice”, but a variety of voices and experiences that need to be considered if policies and practices concerning alternative livelihoods are to be effective. Involving bidi workers in discussions about their circumstances, assets and aspirations enhances the potential for developing viable economic alternatives to tobacco at the grassroots level. Our project demonstrates the scope for greater involvement of health institutions such as the LCECU at CMC Vellore in outreach work of this kind.

## Supplementary Material

A Contributorship Form detailing each author’s specific involvement with this content, as well as any supplementary data, are available online at https://academic.oup.com/ntr.

ntac075_suppl_Supplementary_Taxonomy-form

## Data Availability

The data underlying this article will be shared on reasonable request to the corresponding author.
